# Towards Predicting Immune-Related Adverse Events: Emerging Biomarkers in Patients Undergoing Immune Checkpoint Inhibitor Therapy

**DOI:** 10.3390/cancers18172759

**Published:** 2026-08-25

**Authors:** Nežka Hribernik, Martina Reberšek

**Affiliations:** 1 Department of Medical Oncology, Institute of Oncology Ljubljana, 1000 Ljubljana, Slovenia; 2 Faculty of Medicine, University of Ljubljana, 1000 Ljubljana, Slovenia

**Keywords:** immune-related adverse events, immune-checkpoint inhibitors, predictive biomarkers, gut microbiome, body composition, molecular imaging, multi-omics

## Abstract

With immune checkpoint inhibitors becoming the mainstay of systemic therapy in both metastatic and early-stage settings across many cancer types, the management of immune-related adverse events has emerged as a central priority of modern oncological supportive care. These toxicities can substantially impair the quality of life of cancer patients, including those who achieve long-term survival. Consequently, there is a pressing need to develop reliable predictive biomarkers to better tailor immune checkpoint inhibitor treatment and optimize patient selection. This review summarizes several of the most promising predictive biomarkers currently under investigation, including genetic factors; peripheral blood parameters and their ratios; autoantibodies; cytokines and chemokines; cytomegalovirus serostatus; gut microbiome characteristics; body composition metrics; molecular imaging features; and tumour- and patient-related factors such as cancer type, gender, and physical activity. Because single biomarkers have limited predictive value, multi-omics prediction models and composite immune-cell scores are increasingly demonstrating greater potential. However, none of these candidate biomarkers have yet undergone sufficient validation to support their incorporation into routine clinical practice.

## Simple Summary

Immune-checkpoint inhibitors are one of the main pillars of systemic cancer treatment, used in both radical and metastatic settings. However, they can cause immune-related adverse events that may limit treatment efficacy and negatively affect the quality of life of cancer patients. Identifying biomarkers that predict these toxicities has therefore become a key priority in immune oncology. This review summarizes current evidence on several promising biomarker groups, including genetic and immunologic factors, gut microbiome features, body composition, molecular imaging, and others. Although early results are encouraging, more prospective clinical studies are needed before these biomarkers can be implemented into everyday clinical practice.

## 1. Introduction

Immune-checkpoint inhibitors (ICIs) have become one of the most important systemic cancer treatments in the past decade. For a wide range of solid-organ and hematologic malignancies, ICIs now represent the standard of care [1]. These agents induce immune-mediated destruction of cancer cells by blocking immunosuppressive checkpoint molecules such as cytotoxic T-lymphocyte-associated antigen 4 (CTLA-4), programmed death cell protein 1 (PD-1), programmed death ligand 1 (PD-L1), lymphocyte activation gene 3 (LAG-3), and several others that physiologically restrain the activity of tumour-reactive T cells [2,3,4,5]. Their side effects, known as immune-related adverse events (irAEs), can limit the efficacy of ICIs and negatively affect the quality of life of cancer patients [6]. These off-target manifestations of excessive immune activation may involve virtually any non-malignant tissue. Nevertheless, dermatologic, endocrine, gastrointestinal, hepatic, and pulmonary toxicities are most frequently observed. Current guidelines for the management of irAEs emphasize early recognition of toxicities and prompt initiation of immunosuppressive treatment [4,5,6,7].

The mechanisms underlying irAEs are still being elucidated and likely arise from both tumour-related and tumour-unrelated factors. Tumour-specific mechanisms include neoantigen–host antigen homology, tissue-specific cross-reactivity, and host genetic susceptibility. Non-tumour-specific mechanisms involve the microbiota, cytokine signalling, tissue-intrinsic factors, viral triggers, and antibody-dependent cellular cytotoxicity [8]. Certain mechanisms appear to be more closely associated with specific irAE subtypes. For example, hypophysitis has been linked to enhanced complement-mediated inflammation caused by direct binding of anti-CTLA-4 antibodies to CTLA-4 expressed on normal pituitary cells. The broad heterogeneity of irAEs is therefore likely to reflect distinct—and often highly diverse—mechanistic pathways for each toxicity subtype [8].

The widespread use of ICIs in both metastatic and early-stage settings has led to a growing number of patients experiencing irAEs. Importantly, ICI toxicities may become chronic or permanent, affecting up to 40% of patients long after treatment cessation and substantially impairing quality of life, including among long-term survivors [8].

Predictive and prognostic biomarkers (BM) are measurable biological, molecular, or cellular features that inform clinical decision-making by forecasting disease course or treatment response. Prognostic biomarkers provide information on the likely outcome of a patient—such as recurrence, progression, or death—independent of therapy, reflecting tumour biology and host factors [9]. Predictive biomarkers identify patients most likely to experience a favourable or unfavourable effect from exposure to a medical product or an environmental agent [9]. Both classes of biomarkers are fundamental to precision oncology, enabling personalised treatment selection and avoidance of ineffective or toxic therapies. Their development moreover relies on high-throughput genomic, transcriptomic, and proteomic technologies, in combination with rigorous clinical validation [10]. Some biomarkers may have both prognostic and predictive value, and distinguishing between these roles can occasionally be challenging.

There is a strong need to develop predictive biomarkers for irAE development to better select patients for ICI therapy. Incorporating predictive biomarkers into clinical practice would help clinicians more accurately weigh the risks and benefits of ICIs. Although numerous studies are currently investigating potential predictive biomarkers of irAEs, none have yet been validated for routine clinical decision-making [5,6,7,11].

In this review, we summarise several of the most promising predictive biomarkers currently under investigation. We primarily focus on predictive biomarkers of irAEs, although relevant comments on the prognostic implications of irAEs are included where appropriate.

## 2. Categories of Predictive Biomarkers

### 2.1. Genetic Predictive Biomarkers

A number of studies have identified genetic factors that may predispose cancer patients to developing irAEs. Among these, the Human Leukocyte Antigen (HLA) genotype is one of the most extensively investigated, given its well-established association with susceptibility to immune-mediated diseases and cancer [12]. Importantly, HLA variants indicate only a genetic predisposition rather than a definitive diagnosis of autoimmune disease, and therefore HLA testing is not recommended as part of routine diagnostic evaluation for most autoimmune conditions [13]. The potential association between HLA genotypes and the risk of irAEs was initially explored primarily in the context of endocrine toxicities. In cancer patients who develop ICI-related type 1 diabetes mellitus during treatment, a significant predominance of HLA-DR4 has been observed, with susceptible HLA genotypes present in approximately 65% of affected individuals [14]. Additional organ-specific HLA associations have been reported, including HLA-DRB1*04:05 in patients with immune-related inflammatory arthritis, HLA-DRB1*11:01 in those with immune-related pruritus, and HLA-B*27:05 in patients with immune-related encephalitis, among others [15,16,17].

Beyond HLA, several other genetic polymorphisms have demonstrated potential predictive value in cohort studies. For example, MAPK1 rs3810610 has been associated with irAE risk, although its clinical utility remains strictly investigational and far from implementation in routine practice [18]. Overall, more research—particularly prospective studies conducted in larger and more diverse patient cohorts—is needed to clarify the predictive role of genetic alterations in irAEs development and to determine whether these findings can be translated into clinically meaningful risk stratification tools.

### 2.2. Hematologic Predictive Biomarkers

Blood cell counts and their ratios represent some of the most accessible potential predictive biomarkers, as they are widely available, inexpensive, and straightforward to interpret. However, their specificity—one of the most important characteristics of a clinically useful predictive biomarker—remains limited, and most findings have yet to be validated in large prospective cohorts.

Absolute lymphocyte count (ALC) and its association with irAEs have been evaluated in several retrospective studies, which consistently show that patients with higher ALC values are at increased risk of developing irAEs [19]. In a retrospective review of 167 cancer patients treated with ICIs, individuals with a baseline ALC > 2000/µL had a significantly higher risk of irAEs on multivariate analysis (odds ratio 1.996, *p* < 0.05) [20]. Similarly, a high absolute eosinophil count (AEC) at baseline—or an increase during the first month of treatment—has been linked to a greater likelihood of irAEs, particularly dermatologic and endocrine toxicities [21]. A high neutrophil-to-lymphocyte ratio (NLR), a marker of systemic inflammation, has also been associated with increased irAE risk in a systematic review and meta-analysis (odds ratio 1.046, *p* < 0.026) [22].

Although these hematologic markers and others show promise, their predictive strength remains insufficient to meaningfully influence clinical decision-making regarding ICI initiation [13]. Larger, well-designed prospective studies are needed to determine whether these readily available parameters can be integrated into reliable risk-stratification models for irAEs.

### 2.3. Immunologic Predictive Biomarkers

#### 2.3.1. History of Autoimmune Diseases

Patients with pre-existing autoimmune diseases (AIDs) have traditionally been excluded from clinical trials evaluating ICIs. However, evidence from multiple observational studies clearly demonstrates that individuals with established AIDs develop irAEs more frequently and earlier than those without underlying autoimmune conditions [23]. In a French retrospective cohort of cancer patients with pre-existing AIDs treated with ICIs, autoimmune disease flares occurred in 47% of patients, while other irAEs developed in 42% [24].

The severity of flares and the need for high-dose immunosuppression vary across different autoimmune conditions. Certain organ-specific AIDs, such as thyroiditis and vitiligo, tend to follow a more benign course, whereas systemic rheumatic diseases are typically associated with a higher likelihood of flares and may require more intensive immunosuppressive management [25]. Despite the increased risk, the majority of irAEs in patients with AIDs remain manageable, and ICI therapy can often be continued after appropriate intervention [25].

For these reasons, AIDs should be regarded as a predictive biomarker for irAEs rather than a contraindication to ICI use. Their presence signals the need for careful selection of the ICI regimen, closer monitoring during the early phases of treatment, and clear, pre-defined plans for managing potential flares in collaboration with relevant specialists.

#### 2.3.2. Autoantibodies

The presence of pre-existing autoantibodies represents a latent marker of underlying autoimmunity and has been associated with an increased risk of irAEs in numerous studies, with some autoantibodies predicting organ-specific toxicities and others correlating with irAEs across multiple organ systems [26,27,28,29]. For example, patients with pre-existing anti-thyroglobulin antibodies (TgAbs) and/or anti-thyroid peroxidase antibodies (TPOAbs) have a higher likelihood of developing immune-related thyroiditis, while individuals with rheumatoid factor (RF) positivity are at increased risk of rheumatic irAEs [26,27]. Similarly, the presence of antinuclear antibodies (ANAs), antineutrophil cytoplasmic antibodies (ANCA), or RF prior to ICI initiation has been associated with a higher incidence of irAEs affecting various organs [28].

A large retrospective study further expanded the landscape of autoantibody-based prediction by evaluating pretreatment reactivity against 832 human recombinant protein antigens in 331 metastatic melanoma patients. The analysis identified 47 autoantibodies predictive of irAEs, with the strongest markers including KRT7, RPLP2, UBE2Z, and GPHN. Several autoantibodies associated with irAEs—such as anti-ATG4D, anti-MAGEB4, and anti-IL4R—were also linked to improved survival. In contrast, anti-fibroblast growth factor receptor 1 (FGFR1) antibody positivity predicted a reduced risk of irAEs but was associated with shorter survival. Importantly, the study demonstrated that predictive autoantibody signatures differ between ICI subgroups, supporting distinct mechanisms of toxicity for PD-1- and CTLA-4-based therapies [29].

Although pretreatment autoantibody profiling offers meaningful risk enrichment, routine testing is not currently recommended in clinical practice according to existing guidelines [4,30]. Further prospective validation is required before autoantibody-based prediction can be integrated into standard clinical decision-making.

#### 2.3.3. Cytokine and Chemokine Levels

Several studies have highlighted the relevance of baseline cytokine and chemokine levels, as well as their dynamic fluctuations during treatment, in the development of irAEs. However, despite growing interest, the overall strength of evidence supporting their predictive value remains limited [30]. Lower baseline levels of pro-inflammatory cytokines such as interleukin-6 (IL-6) (odds ratio = 2.84 [95% CI 1.34–6.03], *p* = 0.007) and soluble cluster of differentiation 163 (*p* = 0.0018) have been associated with the occurrence of irAEs in general [31,32]. Elevated baseline levels of interleukin-17 (IL-17) have been specifically linked to the development of high-grade immune-related colitis (*p* = 0.02) [33]. CXCL9 and CXCL10—both interferon-γ (IFN-γ)-induced chemokines involved in the recruitment of activated T cells—were found to be increased in patients who developed irAEs [34]. The roles of many additional cytokines and chemokines remain to be clarified. Overall, these markers represent promising components of emerging multimodal risk-prediction models, where they may contribute to more refined and biologically informed risk scores.

#### 2.3.4. Cytomegalovirus Serostatus

Cytomegalovirus (CMV) is a globally endemic latent herpesvirus that profoundly influences T-cell immunity. The spectrum of CMV-related disease is broad and largely dependent on host immune status. CMV is a major infectious cause of congenital abnormalities and represents an important pathogen in immunocompromised individuals [35].

A prospective study of 341 patients receiving ICIs for melanoma demonstrated a clinically meaningful association between CMV serostatus, response to PD-1 blockade, and irAE incidence [36]. CMV-seropositive melanoma patients exhibited higher lymphocyte counts, reduced NLR, and distinct CD8^+^ T-cell gene-expression profiles. Clinically, CMV-seropositive patients treated with PD-1 inhibitors had improved survival and, unexpectedly, a significantly reduced cumulative incidence of severe irAEs at 6 months (0.30 vs. 0.52; *p* = 2.2 × 10^−5^), including lower rates of immune-related colitis and pneumonitis. When using multivariable analysis to control for the age, sex, ICI type, and treatment setting, both the known reduced toxicity of ICBs and CMV remained independently associated with significantly reduced incidence of severe irAEs (*p* = 0.0058). These findings may be explained by CMV-mediated modulation of tissue immunity, which could alter susceptibility to excessive immune activation during ICI therapy [36].

Taken together, CMV infection may confer partial protection against severe irAEs, underscoring the potential importance of chronic immune activation shaped by prior infections. Future studies incorporating detailed immunophenotyping of relevant tissues—while accounting for CMV serostatus—will be essential to further elucidate these mechanisms.

### 2.4. Gut Microbiome as a Predictive BM

The human gut microbiota and its associated microbial metabolites play crucial roles in host metabolism, inflammation, and immune regulation [4]. These microbial communities influence both local and systemic immune responses. Numerous studies have demonstrated that the gut microbiota affects the efficacy of ICIs in cancer patients, and growing evidence from preclinical and clinical research indicates that it also contributes to the development of irAEs [37]. Mechanisms underlying the association between gut microbiota and irAEs include antigen cross-reactivity, modulation of intestinal barrier integrity, interactions with the immune microenvironment, regulation of microbial homeostasis, and other immunomodulatory pathways [2].

Gut dysbiosis—characterized by reduced microbial diversity and dominance of specific bacterial taxa—has been associated with altered risk of irAE development [37]. A high abundance of Firmicutes has been linked to a significantly increased risk of ICI-related colitis [38]. In another study profiling the gut microbiome of 77 patients with advanced melanoma treated with ICIs, investigators observed a high rate of irAEs and identified distinct microbial signatures: *Bacteroides intestinalis* and *Intestinibacter bartlettii* were enriched in patients who developed irAEs, whereas *Anaerotignum lactatifermentans* and *Dorea formicigenerans* were enriched in those without such toxicities [39].

A comprehensive machine-learning analysis using a random forest approach was performed on microbiome datasets together with in-house 16S rRNA and shotgun metagenomic profiles from 115 patients with irAEs. By integrating transcriptomic and metagenomic data, the study identified specific microbial species capable of distinguishing patients who developed irAEs from those who did not. The random forest classifier, built using 14 microbial features, demonstrated strong discriminatory performance between non-irAE and irAE cases (AUC = 0.88) [40]. Furthermore, anti-CTLA-4 and anti-PD-1/PD-L1 therapies appeared to be associated with distinct microbial biomarkers, suggesting therapy-specific microbiome–immune interactions [40].

Overall, there is not a great deal of overlap between specific bacterial taxa across different published studies [37]. These differences may be related to different factors. Differences in techniques used to analyse samples, reference databases used for analysis, geographical influences as studies are performed in centers at different locations, different dietary and lifestyle factors, effect of comedications, and many others [2].

Concurrent medications that influence gut microbiota composition—such as antibiotics and proton pump inhibitors (PPIs)—also represent potential risk modifiers for irAEs. Studies suggest that the antimicrobial spectrum of antibiotics, timing relative to ICI initiation, and cumulative exposure may all affect irAE risk, although no consensus has yet been reached regarding the overall relationship between antibiotic use and irAEs [2]. Long-term PPI use can induce gut dysbiosis, and a retrospective study reported that lower gastrointestinal irAEs were more severe among PPI users, who experienced higher hospitalization rates and longer hospital stays [41,42]. A recent single-centre retrospective study evaluated 1228 patients receiving ICIs between January 2010 and February 2024 who developed upper or lower gastrointestinal irAEs, stratifying them according to PPI use. Upper gastrointestinal irAEs were more severe among PPI users (69.6% grade 3 toxicity vs. 29.6% in non-users; *p* < 0.05). Similarly, lower gastrointestinal irAEs were more severe in PPI users, who required more frequent use of multiple biologic therapies and had higher hospitalization rates and longer hospital stays (*p* < 0.05 for all). PPI use was also associated with significantly worse overall survival among patients treated with ICIs (*p* < 0.05) [41].

A detailed understanding of the microbiome is essential in the era of precision medicine. Identifying the factors that influence gut microbiome composition and developing strategies to modulate it represent key initial steps toward translating microbiome-based insights into clinical applications.

### 2.5. Patient’s Body Composition

Body composition has emerged as an important determinant of outcomes in oncology, reflecting the complex interplay between adipose tissue, skeletal muscle, systemic inflammation, and host immunocompetence. Among its key components, obesity and sarcopenia represent two distinct but clinically relevant phenotypes frequently encountered in cancer patients [43].

#### 2.5.1. Obesity

Obesity is characterized by excess adiposity and a chronic low-grade inflammatory state. Several studies have reported an association between obesity and improved responses to ICIs, although the underlying mechanisms remain incompletely understood. One proposed explanation is that obesity induces leptin-mediated T-cell dysfunction, which may be reversed by ICI therapy [44].

Evidence regarding the relationship between obesity and irAEs is more heterogeneous. Some, but not all, studies suggest that obesity may increase the risk of irAEs [45]. The chronic pro-inflammatory milieu of adipose tissue likely contributes to an immune environment primed for more pronounced activation upon ICI exposure, potentially influencing both treatment efficacy and toxicity. Obese or overweight patients with a body mass index (BMI) ≥ 25 kg/m^2^ have been statistically linked to a higher frequency and severity of irAEs (hazard ratio 2.0, [95% CI = 1.2–3.4]) [8,45]. These findings carry important clinical implications, as BMI may serve as a simple and accessible factor for baseline risk stratification for irAE development.

#### 2.5.2. Sarcopenia/Cachexia Syndrome

Skeletal muscle is the largest organ in the human body, accounting for approximately 40–50% of total body mass, and plays a central role in whole-body metabolic and functional health [46]. Sarcopenia is a multifactorial syndrome characterized by the loss of skeletal muscle mass and function. It is a well-established poor prognostic factor in patients receiving chemotherapy, targeted therapy, or undergoing surgery. Sarcopenia frequently co-occurs with cachexia, a complex metabolic syndrome marked by severe weight loss, depletion of both fat and muscle, and increased protein catabolism driven by underlying disease [47].

Growing evidence indicates that sarcopenia/cachexia is also a negative prognostic factor in the context of immunotherapy, likely reflecting impaired global immunocompetence that influences both treatment response and toxicity. Importantly, sarcopenia appears to predispose patients to the development of irAEs. Although the evidence is not yet fully uniform, several studies support a meaningful association. For example, one study reported that patients with metastatic melanoma and baseline sarcopenia were more likely to experience severe irAEs during ipilimumab therapy [48]. Similarly, a retrospective cohort study in advanced or recurrent non-small cell lung cancer (NSCLC) treated with ICI monotherapy found that lower psoas muscle index (PMI), used as a surrogate marker for sarcopenia, was associated with a higher risk of severe irAEs (odds ratio 1.72 [95% CI = 1.05–2.80]) [49].

These observations suggest that incorporating muscle-related measures into baseline risk stratification—alongside other clinical and biological predictors—may have meaningful clinical utility.

### 2.6. Molecular Imaging as a Predictive Biomarker

Molecular imaging, particularly positron emission tomography/computed tomography (PET/CT), is a valuable non-invasive method for detecting irAEs in patients receiving ICIs. PET/CT enables identification, quantification, and longitudinal monitoring of increased metabolic activity indicative of inflammation across organs such as the gastrointestinal tract, lungs, and thyroid [50,51]. A key advantage of PET/CT is its ability to generate reproducible and quantitative molecular-level data [52].

Recent advances have introduced deep learning-based convolutional neural networks (CNNs) for automated segmentation of metastatic lesions and organs on PET/CT using [^18^F]fluoro-2-deoxy-D-glucose (^18^F-FDG). In a pilot study of 58 metastatic melanoma patients treated with ICIs, a novel quantitative imaging biomarker (QIB) for irAE prediction was proposed: percentiles of the standardized uptake value distribution (SUV*_X_*_%_) of ^18^F-FDG uptake [51]. PET/CT scans of these patients were retrospectively analysed for signs of irAEs. Three target organs most commonly affected by irAEs—lung, bowel, and thyroid—were segmented using a CNN, and SUV*_X_*_%_ values were correlated with irAE occurrence. The area under the receiver-operating characteristic curve (AUROC) quantified irAE detection performance. Patients without irAEs served as the reference group for establishing normal ranges of organ-specific ^18^F-FDG uptake.

A total of 31% of patients experienced irAEs in the three target organs: immune-related colitis (*n* = 6), pneumonitis (*n* = 5), and thyroiditis (*n* = 9). Optimal percentiles for identifying irAEs were bowel (SUV_95%_, AUROC = 0.79), lung (SUV_95%_, AUROC = 0.98), and thyroid (SUV_75%_, AUROC = 0.88). Corresponding optimal cut-offs were bowel (SUV_95%_ > 2.7 g/mL), lung (SUV_95%_ > 1.7 g/mL), and thyroid (SUV_75%_ > 2.1 g/mL). Increased ^18^F-FDG uptake in irAE-affected organs provided predictive information regarding subsequent toxicity development, and in several cases irAEs were detectable on PET/CT before clinical symptoms emerged. These findings suggest that SUV*_X_*_%_ may represent a robust QIB for irAE prediction [51].

Importantly, the diversity of scanners and treatment settings used in the study further strengthened the robustness of the analysis by incorporating factors that could influence SUV percentile calculations. Sensitivity and specificity values were reported for the cut-off that maximized their combined sum; however, alternative operating points may be more appropriate depending on clinical needs. For example, bowel SUV_95%_ demonstrated 100% sensitivity but only 49% specificity, whereas operating points of 83% sensitivity/64% specificity may be preferable in certain clinical contexts [51].

Despite its promise, imaging-based biomarkers have limitations. irAEs can affect virtually any organ, including rare sites such as ocular or cerebral tissues [5,6]. Detecting imaging biomarkers for these uncommon irAEs would require large cohorts. Conversely, some irAEs—such as cutaneous toxicities—are common and readily diagnosed clinically, making imaging unnecessary.

A prospective study of 71 metastatic melanoma patients evaluated the prognostic value of early ^18^F-FDG PET/CT performed at week four after ICI initiation and identified it as a strong prognostic imaging biomarker [53]. Median overall survival differed significantly between patients with clinical benefit (median OS not reached; 95% CI = 17.8 months—NR) and those without clinical benefit (median OS 6.2 months; 95% [CI = 4.6 months—NR]; *p* = 0.003). This study did not include quantitative SUV*_X_*_%_ analysis as a predictive biomarker of irAEs [53].

Beyond ^18^F-FDG, novel radiopharmaceuticals targeting immune-relevant pathways are increasingly being investigated to better understand and predict irAEs [50,54]. Multimodal imaging—integrating nuclear medicine with other imaging modalities—offers complementary information, while multiparametric and multiplex imaging approaches further expand diagnostic capabilities [55]. Advances in probe development, imaging hardware, and data-analysis methods have accelerated progress in this field. Artificial intelligence (AI) and machine learning are transforming radiology, enabling multimodal imaging to leverage large, complex datasets. However, challenges remain, including lack of standardization and complex regulatory requirements [55].

For imaging to have clinical impact, high specificity for irAE-related processes is essential. Total radiation exposure and optimal timing of imaging also pose challenges, as irAEs develop at variable time points. Radiotracers designed to visualize CD8^+^ T-cell localization, activated fibroblasts, and heightened metabolic activity currently show the greatest potential for predicting and monitoring irAEs [50,52]. Overall, molecular imaging holds significant promise for improving mechanistic understanding of irAEs and enhancing their detection.

## 3. Other Nonspecific Predictive Biomarkers

### 3.1. ICI Agent and Treatment Setting

Different ICI agents and treatment regimens have distinct toxicity profiles [8]. The choice between monotherapy and combination therapy is one of the strongest determinants of irAE risk: high-grade irAEs occur in 30–55% of patients receiving combination therapy, compared with only 10–15% of those treated with monotherapy [4]. CTLA-4 antibodies are associated with the highest burden of immune-related colitis/diarrhea, immune-related hypophysitis, and immune-related skin toxicities, whereas PD-(L)1 antibodies are more commonly linked to immune-related thyroiditis, pneumonitis, and hepatitis [56].

The development of irAEs during treatment with curative intent is of particular concern, as these toxicities can be more debilitating—especially when they become chronic or irreversible. Initially, there was apprehension that neoadjuvant ICIs might predispose patients to higher irAE rates, given their ability to induce strong immune activation and improve survival outcomes. However, a meta-analysis in NSCLC patients comparing irAE rates across treatment settings provided reassuring evidence [57]. Data from 15 phase III clinical trials leading to the approval of PD-(L)1 ICIs in neoadjuvant, adjuvant, peri-operative, and metastatic settings showed that irAE rates were lower in neoadjuvant, peri-operative, and adjuvant therapy than in metastatic treatment. A neoadjuvant-only approach was associated with the lowest incidence of irAEs, despite demonstrating superior efficacy [57].

These findings may be explained by several factors, including the lower number of treatment cycles administered in neoadjuvant protocols, patient selection, and the tendency for some irAEs to emerge later—during adjuvant or prolonged therapy rather than during the neoadjuvant phase.

### 3.2. Tumor Type and Its Characteristics

Neither tumour type, mutation burden, nor PD-L1 expression appears to substantially influence the overall incidence of irAEs. However, certain cancer types seem to be associated with specific patterns of irAEs, although the evidence remains weak and some studies have reported conflicting results [4]. One notable exception is skin and hair vitiligoid depigmentation, a form of cutaneous irAE most commonly observed in melanoma patients treated with ICIs, occurring in approximately 5–25% of cases [4]. Vitiligoid depigmentation also represents a positive prognostic biomarker, as several studies have shown that melanoma patients who develop cutaneous irAEs experience significantly longer survival [53]. In a prospective study of metastatic melanoma patients, median overall survival was not reached (95% CI = 23.7 months—NR) in those who developed cutaneous irAEs, compared with 8.2 months (95% CI = 4.6–17.8) in patients without cutaneous irAEs (*p* < 0.0001). Vitiligoid depigmentation reflects an autoimmune response against melanocytes and serves as a reliable indicator of antitumour activity [53,58].

Immune-related pneumonitis has also been reported more frequently in patients with renal cell carcinoma and NSCLC [56]. However, this observation may be influenced by confounding characteristics of these patient populations, such as smoking history, underlying pulmonary disease, or prior treatments.

### 3.3. Patient’s Gender

For decades, gender-based differences in chemotherapy toxicity have been recognized, with women experiencing more adverse events than men [59]. Available data indicate that women are also more susceptible to toxicities from other systemic treatments, including ICIs. A large retrospective study evaluated adverse events by gender across multiple treatment modalities using data from 23,296 patients enrolled in 202 clinical trials between 1989 and 2019. Both symptomatic and objective treatment-related toxicities were assessed. Women receiving immunotherapy had a 49% higher risk of severe irAEs compared with men (odds ratio 1.49 [95% CI = 1.24–1.78]). They also had a 66% higher risk of symptomatic irAEs and were more likely to experience severe hematologic irAEs [60].

These gender-related differences in irAEs likely arise from multiple factors, including subjective differences in symptom reporting, as well as biological variations in pharmacokinetics, pharmacodynamics, and pharmacogenomics. Together, these findings underscore the importance of considering patient gender in irAE risk assessment and monitoring strategies.

### 3.4. Patient’s Age

The use of ICIs is increasing among older adults, a highly heterogeneous population characterized by age-related changes in immune function, a higher prevalence of comorbidities, and varying degrees of frailty. Importantly, older patients have been underrepresented in pivotal clinical trials, leaving gaps in our understanding of ICI safety in this subgroup. Managing irAEs in older adults can be particularly challenging due to frailty, comorbid conditions, and reduced physiological reserve [61,62,63].

A study evaluating irAE occurrence in older adults using the U.S. Food and Drug Administration Adverse Event Reporting System—the largest publicly available repository of unsolicited adverse-event reports—compared irAE incidence and characteristics between older and younger patients. Among 47,513 cancer patients aged 18–100 reporting irAEs, individuals aged 65–74 and 75–84 had significantly increased risks of irAEs compared with those aged 18–64 (odds ratio 1.13 [95% CI = 1.09–1.18] and 1.15 [95% CI = 1.10–1.21], respectively) [63]. Interestingly, serious outcomes slightly decreased in patients older than 85, although the proportion of deaths increased with age. Certain irAE types were more common in older adults, including cardiovascular, renal, and musculoskeletal toxicities, whereas endocrine, hepatobiliary, gastrointestinal, and ocular irAEs were more frequent in younger patients [63].

Data on ICI toxicity in pediatric patients and young adults are similarly limited. In a large retrospective multicentre study of 190 melanoma patients younger than 40 years, the overall incidence of irAEs of any grade was comparable to that observed in older adults. However, immune-related hepatitis occurred at a higher incidence in younger patients [64].

### 3.5. Patient’s Physical Activity

The health benefits of physical activity (PA) are increasingly recognized in oncology [65]. Studies have shown that higher levels of PA in cancer patients receiving chemotherapy improve survival and reduce treatment-related side effects [66]. Similarly, in patients treated with ICIs, PA appears to reduce the occurrence of irAEs. The strongest evidence comes from a prospective observational study evaluating baseline PA before ICI initiation and assessing its association with irAEs and survival. A total of 251 patients receiving ICIs were included, with a median follow-up of 20 months. Moderate and high levels of total PA were associated with significantly lower odds of severe irAEs compared with low PA levels (adjusted odds ratio 0.34 [95% CI = 0.12–0.90] and 0.19 [95% CI = 0.05–0.55], respectively). Moderate and high PA levels were also associated with prolonged survival [67].

The mechanisms by which exercise may reduce autoimmunity and enhance treatment effectiveness are not yet fully understood. Insights from exercise immunology suggest that physical activity modulates cytokine profiles, increasing interleukin-10 (IL-10) and reducing chronic IL-6/TNF-α signalling, thereby shifting the immune system toward a more regulated, anti-inflammatory baseline. Exercise-induced mobilization of regulatory immune cells may buffer excessive immune activation triggered by ICIs. Additionally, reduced systemic inflammation and improved tissue resilience may lower organ susceptibility to autoimmune-like injury [65].

In conclusion, higher physical activity predicts a lower likelihood of severe irAEs. However, whether patients truly benefit from increasing PA levels after diagnosis remains an important question for future randomized controlled trials.

Some examples of possible negative and possible positive predictive biomarkers of irAEs are shown in Table 1 and Table 2, respectively.

## 4. Prediction Models

Prediction models for irAEs remain an important unmet need. Because single biomarkers (Table 1 and Table 2) have shown limited predictive value, current research is increasingly focused on integrative approaches that combine clinical, genetic, immunologic, and microbiome data. However, most available evidence still derives from retrospective analyses, limiting the strength and generalizability of current predictive signatures.

A large multi-omics retrospective study of more than 18,000 patients with 26 cancer types treated with ICIs evaluated predictive models for irAEs by integrating pharmacovigilance and molecular datasets. Among six factors associated with irAE risk, T-cell receptor (TCR) diversity and CD8^+^ T-cell abundance demonstrated the strongest correlations. Gene expression of lymphocyte cytosolic protein 1 (LCP1) and adenosine-diphosphate–dependent glucokinase (ADPGK) also showed high predictive potential, although prospective validation is required before clinical implementation [68]. Composite T-cell biomarker scores—including effector-memory CD4^+^ T-cell frequency and TCR clonality in pre-treatment blood—have similarly correlated with irAE risk, but larger cohorts are needed to confirm these associations [69]. High-dimensional immune profiling, such as T follicular helper (Tfh) cell signatures and inflammatory protein panels including IL-12β, IL-15RA, and CXCL9, may further refine risk prediction [70].

Despite these advances, external validation remains challenging. In a cohort of 110 patients treated with nivolumab plus ipilimumab, external testing of 59 previously proposed biomarkers showed poor discriminatory performance, and even advanced clustering of T-cell subsets did not yield reliable classifiers. Flow cytometry and clinical data alone were insufficient to reliably predict irAE risk in most cases [71]. Recent reviews and meta-analyses highlight cytokines (e.g., IL-6, IL-17), autoantibodies, and blood cell ratios as promising candidates, but most studies remain retrospective, single-center, or limited to specific cancer types or irAE subtypes [72,73]. Composite immune signatures and microbiome–immune integration approaches are emerging, but remain far from clinical application and require prospective, multi-center validation [2].

Several ongoing clinical trials are now prospectively incorporating biomarker discovery and validation for irAE prediction, including protocols with serial blood sampling, high-dimensional immune profiling, and microbiome analyses in both neoadjuvant and metastatic ICI settings [72]. These prospective efforts are essential for establishing reproducible, clinically actionable biomarkers and may help bridge the gap between retrospective signal-finding and real-world implementation.

Also, these potential prediction models have not undergone regulatory validation or multicentre harmonization. Although they perform well in retrospective cohorts, they remain vulnerable to dataset bias, overfitting, limited ethnic diversity, and the absence of external validation, all of which restrict their diagnostic applicability [10]. Another prerequisite for their implementation is the need for robust bioinformatics infrastructure, including standardized pipelines for data processing, model training, and quality control [10]. Moreover, complex models must be made more accessible and usable in real-world clinical settings, requiring user-friendly interfaces, transparent reporting standards, and integration with existing clinical workflows.

Taken together, current prediction models for irAEs show promise but remain in an early developmental stage. Substantial methodological refinement, rigorous external validation, and harmonization across institutions will be required before these tools can be reliably deployed in routine oncology practice.

## 5. Conclusions

ICIs represent one of the main pillars of modern systemic cancer therapy but can cause acute or chronic irAEs that may significantly impair daily functioning and, in some cases, lead to debilitating long-term consequences. Numerous potential predictive biomarkers are under investigation, and several show promise for anticipating irAE risk. However, none are currently recommended for routine use in daily clinical practice. Multi-omics prediction models and composite immune-cell scores are among the most promising approaches, yet they still require prospective validation before integration into standard care [3,13]. Novel AI methodologies may further enhance prediction modelling by integrating heterogeneous datasets, including clinical, imaging, multi-omics, and microbiome data.

Importantly, biomarker implementation follows a multi-step pathway—from technical validation and infrastructure development to reimbursement, funding considerations, clinician education, and guideline harmonization. Predictive biomarkers for irAEs therefore remain on the horizon, awaiting robust evidence and structured pathways that will enable their translation into real-world clinical practice.

## Figures and Tables

**Table 1:** Some examples of possible negative predictive biomarkers of irAEs.

Biomarker Category	Possible Specific Biomarker	Reduced Risk for irAEs
Autoantibodies	Anti-FGFR1 antibody	Reduced risk of irAEs in general
Infections	CMV seropositivity	Reduced risk of immune-related colitis and pneumonitis
Microbiota	Elevated *Anaerotignum lactatifermentans*	Reduced risk of irAEs in general
ICIs agent	Mono-ICIs (vs. combination)	Reduced risk of irAEs in general
Treatment setting	Neoadjuvant ICIs	Reduced risk of irAEs in general
Gender	Male sex	Reduced risk of irAEs in general
Physical activity	Higher baseline physical activity	Reduced risk in severe irAEs

Abbreviations: CMV: cytomegalovirus; FGFR1: fibroblast growth factor receptor 1; ICIs: immune-checkpoint inhibitors; irAEs: immune-related adverse events.

**Table 2:** Some examples of possible positive predictive biomarkers of irAEs.

Biomarker Category	Possible Specific Biomarker	Predicted irAEs
Genetic	HLA-DR4	Immune-related diabetes mellitus type 1
Hematological	Elevated Eosinophils	Immune-related skin and endocrine toxicity
Autoantibodies	TPOAb/TgAb	Immune-related thyroiditis
Cytokines	Elevated IL-17	High-grade immune-related colitis
Gut microbiome	Elevated *Firmicutes*	Immune-related colitis
Concurrent medications	Proton pump inhibitors	High-grade immune-related colitis
Body composition	Obesity, sarcopenia	Higher frequency and severity of irAEs
Cancer type	Melanoma	Skin and hair vitiligoid depigmentation
Molecular imaging	SUV*_X_*_%_ of ^18^F-FDG uptake	Immune-related colitis, thyroiditis, pneumonitis

Abbreviations: ^18^F-FDG: [^18^F] fluoro-2-deoxy-D-glucose; HLA: human leukocyte antigen; IL: interleukin; irAEs: immune-related adverse events; SUV*_X_*_%_: percentiles of SUV distribution; TgAb: anti-thyroglobulin antibody; TPOAb: anti-thyroid peroxidase antibody.

## Data Availability

This study is a narrative review and does not contain original experimental data. All information synthesized and referenced is sourced from the publicly available literature as cited throughout the manuscript.

## Abbreviations

The following abbreviations are used in this manuscript:

ADPGK	Adenosine diphosphate-dependent glucokinase
AEC	Absolute eosinophil count
AI	Artificial intelligence
AIDs	Autoimmune diseases
ALC	Absolute lymphocyte counts
ANA	Antinuclear antibody
ANCA	Antineutrophil cytoplasmic antibody
AUC	Area under the curve
AUROC	Area under the receiver-operating characteristic curve
BM	Biomarker
BMI	Body mass index
CI	Confidence interval
CNN	Convolutional neural network
CMV	Cytomegalovirus
CTLA-4	Cytotoxic T lymphocyte-associated antigen 4
^18^F-FDG	[^18^F]fluoro-2-deoxy-D-glucose
FGFR1	Fibroblast growth factor receptor 1
HLA	Human leukocyte antigen
ICIs	Immune-checkpoint inhibitors
IFN-γ	Interferon gamma
IL	Interleukin
irAEs	Immune-related adverse events
LAG-3	Lymphocyte activation gene 3
LCP1	Lymphocyte cytosolic protein 1
NLR	Neutrophil-to-lymphocyte ratio
NSCLC	non-small cell lung cancer
PA	Physical activity
PD-1	Programmed death cell protein 1
PD-L1	Programmed death ligand 1
PET/CT	Positron emission tomography
PMI	Psoas muscle index
PPI	Proton pump inhibitors
RF	Rheumatoid factor
SUV*_X_*_%_	Percentiles of SUV distribution
TCR	T-cell receptor
TgAbs	Anti-thyroglobulin antibodies
TNF-α	Tumor necrosis factor α
TPOAbs	Anti-thyroid peroxidase antibodies
Tregs	Regulatory T-cells
QIB	Quantitative imaging biomarker
